# Individual Restriction Of Fine Specificity Variability In Anti-GM1 IgG Antibodies Associated With Guillain-Barré Syndrome

**DOI:** 10.1038/srep19901

**Published:** 2016-01-28

**Authors:** Ricardo D. Lardone, Nobuhiro Yuki, Fernando J. Irazoqui, Gustavo A. Nores

**Affiliations:** 1 Departamento de Química Biológica “Dr. Ranwel Caputto” - CIQUIBIC, CONICET, Facultad de Ciencias Químicas, Universidad Nacional de Córdoba, Córdoba, Argentina; 2 Brain and Mind Centre, University of Sydney, Sydney, Australia

## Abstract

Elevated titers of serum antibodies against GM1 ganglioside are associated with a variety of autoimmune neuropathies. Much evidence indicates these autoantibodies play a primary role in the disease processes, but the mechanism for their appearance is unclear. We studied the fine specificity of anti-GM1 antibodies of the IgG isotype present in sera from patients with Guillain-Barré syndrome (GBS), using thin-layer chromatogram-immunostaining of GM1, asialo-GM1 (GA1), GD1b and GM1-derivatives with small modifications on the oligosaccharide moiety. We were able to distinguish populations of antibodies with different fine specificity. Remarkably, individual patients presented only one or two of them, and different patients had different populations. This restriction in the variability of antibody populations suggests that the appearance of the anti-GM1 antibodies is a random process involving restricted populations of lymphocytes. With the origin of disease-associated anti-GM1 antibodies as a context, this finding could provide explanation for the “host susceptibility factor” observed in GBS following enteritis with GM1 oligosaccharide-carrying strains of *Campylobacter jejuni*.

Antibodies that recognize gangliosides (sialic acid-containing glycolipids found abundantly in nervous tissues) have been associated with different neuropathies^1^,^2^,^3^. Ganglioside GM1 is one of the best-studied antigenic targets and can be considered a model of the association between anti-ganglioside antibodies and disease. Although a large body of cumulative data indicates anti-GM1 antibodies play a primary role in Guillain-Barré syndrome (GBS) pathophysiology (for review^4^), less information is available on the origin of the antibodies. GM1 ganglioside is a self-antigen, and consequently its immune response should be restricted by self-tolerance^5^. Low affinity IgM antibodies reacting with GM1 are part of the normal repertoire of human antibodies^6^. In contrast, patient anti-GM1 antibodies have higher affinity^7^ or different isotype^8^,^9^. The spectrum of diseases described to be associated with anti-GM1 antibodies is wide, but it is possible to find some distinctions regarding antibody isotype. IgM antibodies are mainly associated with chronic diseases, whereas IgG antibodies are typically found in acute forms. Considering that T-cell cooperation is necessary for IgG antibodies induction (while it is not required for induction of the IgM isotype), different antibody-inducing mechanisms could be acting in acute or chronic diseases. Cross-reactivity with glycan antigens of bacteria that colonize the human body is proposed as the origin of normally occurring IgM antibodies^10^. Disease-associated IgM antibodies are characterized by variable and restricted patterns of antibody populations among the different patients^7^. Based on this fact we proposed that disease-associated IgM antibodies originate by random modifications of the binding site of naturally-occurring antibodies (“binding site drift hypothesis”^11^). On the other hand, “molecular mimicry” between a *Campylobacter jejuni* glycan and GM1 has been clearly demonstrated, and is considered the origin of anti-GM1 IgG antibodies found in GBS patients (for review see^12^).

In this paper, we describe a restricted variability in fine specificity of anti-GM1 IgG antibodies from GBS patients. Thus, similarly to the already observed phenomenon for disease-associated anti-GM1 IgM antibodies, these results suggest that the “binding site drift” mechanism could also be contributing to the induction of anti-GM1 antibodies of the IgG isotype.

## Results

### GBS patients’ sera display different anti-GM1 IgG antibody populations

**Figure 1:**
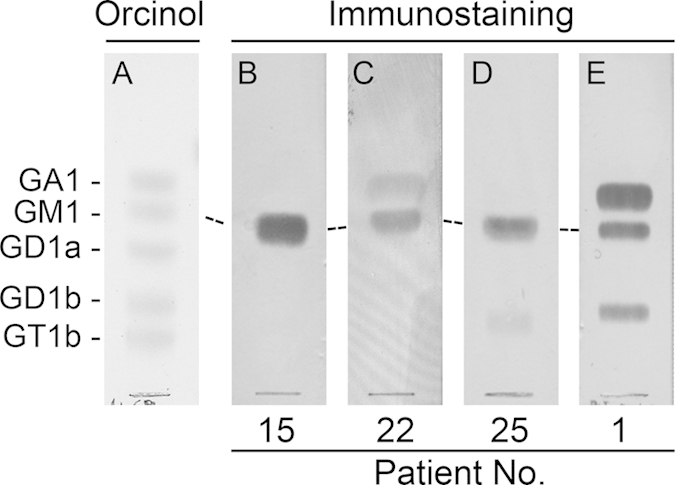
Anti-GM1 IgG immunostaining patterns of patient sera. A mixture of GA1, GM1, GD1a, GD1b and GT1b gangliosides was separated on thin-layer chromatogram plates and immunostained with a 1/200 dilution of sera as described in “Methods”. Representative examples of sera showing reactivity exclusively with GM1 (**B**), or sera showing an additional reactivity with GA1 (**C**), GD1b (**D**) or with both gangliosides (**E**) are shown. A plate was stained with orcinol reagent for chemical detection of gangliosides (**A**).

Thirty GBS sera having anti-GM1 IgG antibodies were selected for this study. Specificity of patient antibodies was assessed by thin-layer chromatography (TLC)-immunostaining and soluble antigen-binding inhibition assay (SABIA). A full summary of serum antibody cross-reactivities and clinical features of GBS patients is shown in Table 1. Antibodies that recognize GM1 can have four different fine specificities, depending if they cross-react or not with two structurally related glycolipids: GA1, desialylated form of GM1; and GD1b, a GM1 molecule with an additional sialic acid residue^7^,^13^. TLC-immunostaining patterns of patient sera were variable. Four representative cases are shown in Fig. 1. Almost half (13) of the sera stained only GM1 (Fig. 1B), whereas the rest also showed cross-reactivity with GA1 (Fig. 1C), GD1b (Fig. 1D) or with both glycolipids (Fig. 1E).

### Fine specificity variability of anti-GM1 IgG antibody populations is restricted within each individual GBS patient

**Figure 2:**
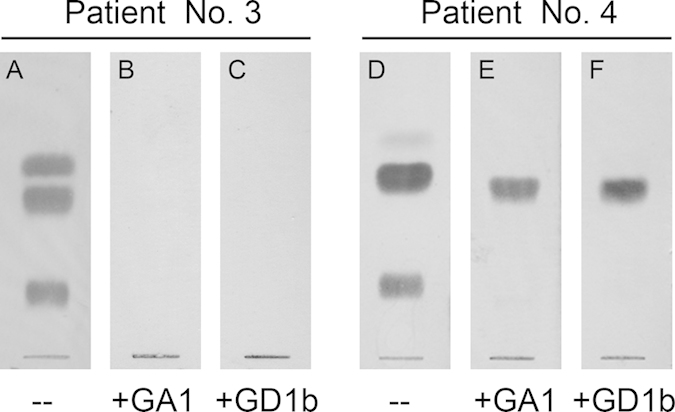
Characterization of anti-GM1 antibody populations of patient sera. Two hundred dilutions of patient sera showing immunoreactivity with GM1, GA1 and GD1b were preincubated without (**A,D**) or with soluble GA1 (**B,E**) or GD1b (**C,F**) at a final concentration of 10^−4^ M. After 1 h sera were used for thin-layer chromatography with immunostaining. Two representative sera exhibiting only one (#3) or two (#4) populations of antibodies are shown.

In all GBS patients, preincubation of sera with soluble GM1 inhibited the binding of anti-GM1 IgG antibodies to TLC-adsorbed GM1 but also to GA1 and GD1b (results not shown), indicating that cross-reacting anti-GM1 antibodies are involved in the staining of GA1 and GD1b. It is clear that sera showing reactivity only with GM1 contained only one antibody population defined by fine specificity (GM1-specific), but sera having cross-reacting antibodies can have more than one population. From twelve sera showing cross-reactivity with both GA1 and GD1b, six contained only one population ~ binding to all three glycolipids (Fig. 2A) was inhibited by preincubation with either GA1 (Fig. 2B) or GD1b (Fig. 2C). In the other six sera, binding to GM1 was not completely inhibited by GA1 (Fig. 2E) or by GD1b (Fig. 2F) indicating that, in addition to cross-reacting antibodies, the sera contained also the GM1-specific population.

The remaining sera showed only one type of cross-reactivity: three of them cross-reacted only with GA1 and two only with GD1b (see Fig. 1C,D). In all sera reacting with GA1, binding to GM1 was completely inhibited by soluble GA1, indicating only one population of antibodies (result not shown). In contrast, both sera cross-reacting exclusively with GD1b contained also a GM1 specific population (results not shown).

**Figure 3:**
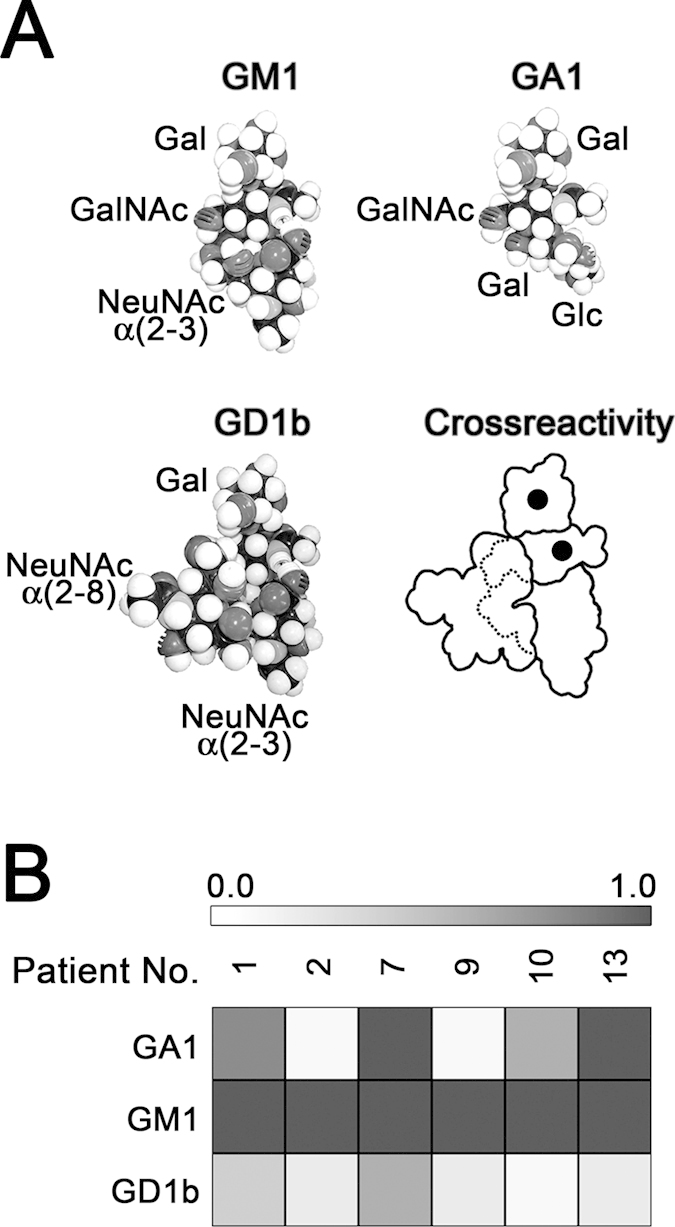
Variability of immunostaining pattern in patient´s sera with cross-reactive anti-GM1 antibodies. (**A**) Axial view (“end side” face) of a Corey-Pauling-Kortum (CPK) model of GA1, GM1 and GD1b oligosaccharides, and their schematic representation. The different models were constructed using torsion angles as described by Acquotti *et al.*^30^,^31^. Black spots on scheme indicate crossreactivity areas (accessible to antibodies in all three structures). (**B**) Seven patient sera having only the population of anti-GM1 antibodies cross-reacting with GA1 and GD1b were analyzed by TLC immunostaining. Spot intensity was measured by densitometric scanning and the O.D. values for GA1 and GD1b were normalized to the O.D. value of GM1 within each patient’s serum.

Although four different populations of anti-GM1 antibodies can be clearly distinguished according to their cross-reactivity with GA1 and GD1b, some additional heterogeneity was observed within these populations. The six sera containing only the population that cross-reacted with GA1/GD1b (Fig. 3A) presented different staining patterns (Fig. 3B): from a serum showing similar cross-reactivity for both glycolipids, to a serum preferentially cross-reacting with one of them.

### Anti-GM1 specific IgG antibodies vary their structural requirements between different GBS patients

**Figure 4:**
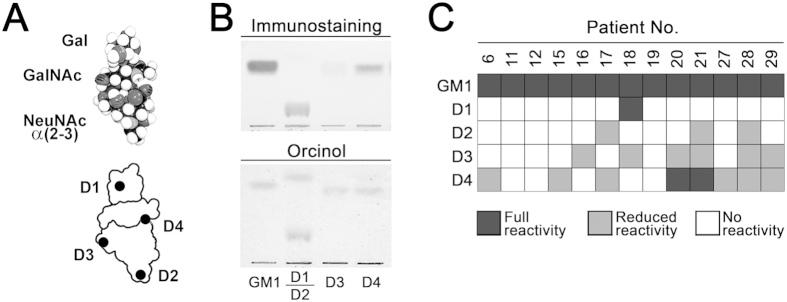
Variability of immunostaining pattern for GM1-derivatives of patient sera with anti-GM1 specific antibodies. (**A**) Axial view of a CPK model of GM1 oligosaccharide, and its schematic representation. Black spots on scheme indicate the area where chemical modification in the GM1-derivatives were present: oxidation/oxime formation of the C6 of the terminal galactose (D1), deacetylation of *N*-acetyl neuraminic acid (NeuNAc) (D2), cleavage of the glycerol chain of NeuNAc (D3) and reduction of the carboxyl group of NeuNAc (D4). (**B**) Reactivity of a patient serum with chemically modified GM1. GM1-derivatives were immunostained with a patient serum having specific anti-GM1 antibodies. One plate was stained with orcinol reagent for chemical visualization of the derivatives. (**C**) Thirteen patient sera having reactivity only with GM1 (not GA1 or GD1b) were used for TLC-immunostaining of GM1-derivatives. Reactivity within each serum was expressed as full, reduced or no reactivity compared to the corresponding anti-GM1 reactivity.

To study the antibody population specific for GM1 in more detail, chemically modified GM1 molecules were used as antigen (Fig. 4A). As exemplified in Fig. 4B, the chemical modification of certain functional groups in the GM1 molecule reduced partially or completely the binding of patient antibodies. Binding to GM1-derivatives was inhibited by preincubation of the sera with soluble GM1, indicating that the same antibodies are involved in the binding to both, the derivatives and the unmodified GM1 (results not shown). Different immunoreactivity patterns with the derivatives were found. Although some patients showed similar results, the patterns of reactivity with the derivatives were quite variable among the different sera (Fig. 4C).

## Discussion

When sera from GBS patients were analyzed by TLC-immunostaining using GM1, GA1 and GD1b gangliosides as antigen, an interesting observation was done: immunostaining pattern was quite different among patients. A further characterization of the antibodies showed a remarkable result: patients had a restricted variability in antibody populations defined by fine specificity. From all different antibody populations we were able to distinguish, individual patients presented only one or two of them, and different patients had different populations. The meaning of this restriction can be analyzed by considering the structure of the recognized antigen. The oligosaccharide moiety of GA1 is included in the structure of GM1, and that of GM1 in GD1b. Based on this fact, the different cross-reactivity of anti-GM1 antibody populations can be explained by the recognition of different areas of GM1 oligosaccharide by the antibodies^7^. A similar interpretation can be done if we analyze the reactivity of GM1-specific antibodies (Fig. 4C) in the context of the GM1 three-dimensional structure (shown in Fig. 4A). Areas differentially recognized account for different antibody binding site structures and, consequently, different B-lymphocyte clones. Therefore, restriction of antibody population variability would indicate that one or few B-lymphocyte clones are involved in each patient´s immune response. This idea implies that the resultant antibodies produced will be monoclonal or oligoclonal, a fact that is known to occur in autoimmune diseases^14^,^15^,^16^. This has been recently described for neuropathy-associated anti-GM1 antibodies through immunoglobulin light chain usage detection^17^. In addition, by affinity purification and further isoelectric focusing of anti-GM1 IgG antibodies from three GBS patients, Townson *et al.* depicted an oligoclonal type of response^18^.

**Figure 5:**
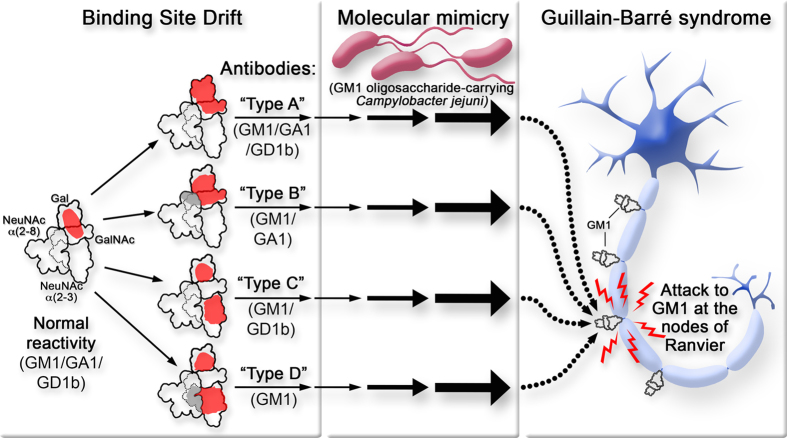
Generation of Guillain-Barré syndrome-associated anti-GM1 IgG antibodies with individual restriction of fine specificity variability in the context of “binding site drift” and “molecular mimicry” mechanisms. B cells producing normally occurring anti-GM1 antibodies (“normal reactivity”) can undergo spontaneous mutations of V genes, randomly re-shaping their binding sites (“binding site drift”). This can lead to eventual increase in binding affinity for GM1 and also to various potential paths generating antibody populations with distinct fine specificity (represented as shaded areas in the different “types”). If only a few lymphocytes get involved, a restricted pattern of fine specificity is produced. During an infection with GM1 oligosaccharide-carrying bacteria (“molecular mimicry”), those diarrheal patients that experienced the “drift” process will get anti-GM1 antibodies with restricted fine specificity induced, and Guillain-Barré syndrome will be triggered.

Most of the patients studied here had a preceding diarrhea, an indication that the “molecular mimicry” mechanism was involved in the generation of antibodies. On the other hand, immunization of rabbits with GM1 in a proper adjuvant induces a classic polyclonal antibody response, including isotype changes and presence of different anti-GM1 antibody populations^13^,^19^. Consequently, the induction of a classical immune response without population restriction would be expected from the “molecular mimicry” mechanism. At this point, a question emerges: why did this not occur? One possible answer is provided by the “binding site drift” hypothesis^11^. This hypothesis was developed to explain the origin of disease-associated anti-GM1 IgM antibodies present in patients with neuropathies^7^. It is based in three facts: i. GM1 is a self-antigen and consequently B-cell clones recognizing GM1 with high affinity should not be present in normal individuals; ii. IgM antibodies that recognize GM1 with low affinity and a defined fine specificity are part of the normal human repertoire of anti-bacterial antibodies; and iii. Disease-associated IgM antibodies have higher affinity for GM1, and show restricted variability in fine specificity. The hypothesis proposes that patient B-cell clones originate from normally occurring ones (Fig. 5). B-lymphocytes producing normal anti-GM1 antibodies spontaneously mutate their V genes, thus modifying their binding sites. Some of these mutations increase the binding affinity for GM1, and the new B-lymphocytes can now be stimulated by self or foreign GM1. During the process, fine specificity can change and various potential paths can be followed, generating antibody populations with distinct fine specificity. Each lymphocyte follows one of these paths at random (“drift”) and if only one or a few lymphocytes are involved, a restricted pattern of populations will be generated. Before or during the infection, in those diarrheal patients where normally occurring B cell clones undergo the “drift” process, antibodies with restricted fine specificity will be induced. If the process occurs in only a few patients, this could explain why only a minority of patients infected with GM1 oligosaccharide-carrying strains of *C. jejuni* develop GBS^20^.

In summary, the emergence of restricted patterns for anti-GM1 antibody populations through events described by the “binding site drift” hypothesis can account for the puzzling “host susceptibility factor” frequently observed in close association with the “molecular mimicry” mechanism in GBS^12^.

## Methods

### Patients

Sera from 30 GBS patients carrying anti-GM1 IgG antibodies were collected at Dokkyo Medical University, Tochigi, Japan, with prior approval from its Ethics Committee. Written informed consent was obtained from every patient. Serum samples taken during the first three weeks after the disease onset, before immune treatment, were stored at –80 °C until use. Sera were analyzed at the Argentinean laboratory. For this purpose, small volume of sera were lyophilized and transported by courier service. Previous experiments done with human and rabbit sera indicated that this treatment does not modify antibody activity (titer, affinity and fine specificity) of anti-ganglioside antibodies. All procedures were approved by the Ethics Committee of CIQUIBIC-CONICET. Criteria for inclusion were a positive spot for GM1 or GM1 and GA1 / GD1b in thin-layer chromatography (TLC)-immunostaining at 1/200 dilution, and no spot for other gangliosides. Twenty-four patients (80%) had diarrhea episodes preceding neurological symptoms. All experiments were performed in accordance with Ethical Guidelines on Research Involving Human Subjects^21^.

### Glycolipids

GM1, GD1a, GD1b and GT1b were obtained from human brain. Folch upper phase was purified by DEAE-chromatography^22^, and HPLC on Iatrobeads silica gel column^23^. GA1 was prepared by acid hydrolysis of cow brain gangliosides^24^.

### GM1 derivatives

D1: Specific modification of C6 of GM1 terminal galactose was done by enzymatic treatment (galactose oxidase) and oxime formation. Briefly, 2.5 mg of GM1 was dissolved in 1 ml of 50 mM sodium phosphate, pH 7.0, containing 1% Triton X-100 and incubated for 24 h. with 15 U of galactose oxidase (Sigma, St. Louis, MO) at 37 °C. The aldehyde product was desalted by a Sep-Pak C18 cartridge (Millipore Corp., Milford, MA) and purified by HPLC^23^. For oxime formation, the purified GM1-aldehyde was dried and dissolved in 1 ml of saturated hydroxylamine hydrochloride in pyridine. After 24 h at RT, 2 ml of water was added and the resulting oxime of GM1 was purified by a Sep-Pak C18 cartridge.

D2: De-*N*-acetylation of GM1 sialic acid was done by mild alkaline hydrolysis in aqueous 90% *N*-butanol^25^.

D3: The glycerol chain of GM1 sialic acid was oxidized to the 7- and 8-aldehyde forms by mild periodation and then reduced to the corresponding truncated primary alcohols as described by Spiegel *et al.*^26^.

D4: Reduction of GM1-sialic acid to the corresponding GM1-nonulosamine (gangliosidol) was accomplished by lactone formation and sodium borohydride treatment^27^.

All GM1 derivatives were further purified by HPLC.

### TLC-immunostaining

Antibody binding to TLC-adsorbed glycolipids was assayed as previously described^28^. Gangliosides were separated on TLC plates in running solvent chloroform/ methanol/ aqueous 0.2% CaCl_2_ (45:45:10), using a tank designed to obtain highly reproducible chromatograms^29^. After air-drying, plates were coated by dipping for 2 min in a 0.5% solution of polyisobutylmethacrylate (Sigma, St. Louis, MO) in *n*-hexane/ chloroform (9:1). Plates were blocked with 1% bovine serum albumin (BSA) in phosphate-buffered saline containing 0.05% Tween 20 (PBSt) for 1 h, incubated overnight with BSA-PBSt diluted serum (1/200), and washed thoroughly with PBSt. Binding was detected following 2 h incubation with BSA-PBSt diluted (1/1000) peroxidase-conjugated anti-human IgG (γ-chain) antibodies (Sigma). All incubation steps were performed at 4 °C. After washing, color was developed in a substrate solution containing 2.8 mM 4-chloro-1-naphthol and 0.01% H_2_O_2_ in methanol-20 mM Tris-HCl buffer, pH 7.4 (1:30). The reaction was stopped after 20 min by washing the plates with PBSt. For quantitative studies, spots were measured by densitometry scanning at 590 λ.

### Soluble antigen-binding inhibition assay

Inhibition of antibody binding to plate bound ganglioside antigen was accomplished by incubating the sera with 0.1 mM GM1, GA1 or GD1b for 60 min before adding them to the plates.

## Additional Information

**How to cite this article**: Lardone, R. D. *et al.* Individual Restriction of Fine Specificity Variability in Anti-GM1 IgG Antibodies Associated with Guillain-Barré Syndrome. *Sci. Rep.* **6**, 19901; doi: 10.1038/srep19901 (2016).

## Figures and Tables

**Table 1:** Serum antibody cross-reactivities and clinical features of Guillain-Barré syndrome patients. R, reactive.

Patient No.	Age	Sex	Precedent diarrhea	Hughes’ functional grade	GM1-specific	GM1-cross-reactive with GA1	GM1-cross-reactive with GD1b
1	32	F	+	2	R	R	R
2	16	F	—	2	—	R	R
3	52	M	+	2	—	R	R
4	26	M	+	2	R	R	R
5	61	M	+	2	—	R	R
6	51	M	+	2	R	—	—
7	38	F	—	3	—	R	R
8	60	M	+	3	—	R	—
9	33	M	+	3	—	R	R
10	33	M	—	3	—	R	R
11	47	M	+	3	R	—	—
12	36	M	+	3	R	—	—
13	12	F	+	4	R	R	R
14	35	F	—	4	R	R	R
15	58	F	+	4	R	—	—
16	53	F	+	4	R	—	—
17	31	F	+	4	R	—	—
18	76	F	+	4	R	—	—
19	40	F	+	4	R	—	—
20	5	F	+	4	R	—	—
21	35	F	+	4	R	—	—
22	18	M	—	4	—	R	—
23	26	M	+	4	—	R	—
24	15	M	+	4	R	R	R
25	12	M	+	4	—	—	R
26	9	M	+	4	R	R	R
27	59	M	+	4	R	—	—
28	61	M	+	4	R	—	—
29	64	M	—	4	R	—	—
30	59	M	+	5	—	—	R
